# From Waste to Function: Compatibilized r-PET/r-HDPE Blends for Pellet Extrusion 3D Printing

**DOI:** 10.3390/polym17121638

**Published:** 2025-06-12

**Authors:** Seyed Amir Ali Bozorgnia Tabary, Jean-Pierre Bresse, Haniyeh (Ramona) Fayazfar

**Affiliations:** Eco-Friendly Circular Advanced Materials and Additive Manufacturing Lab, Department of Mechanical and Manufacturing Engineering, Ontario Tech University, Oshawa, ON L1G 0C5, Canada; seyedamirali.bozorgniatabary@ontariotechu.net (S.A.A.B.T.); jeanpierre.bresse@ontariotechu.net (J.-P.B.)

**Keywords:** recycling, compatibilization, additive manufacturing, circular economy

## Abstract

The increasing accumulation of plastic waste—especially from packaging and post-consumer sources—calls for the development of sustainable recycling strategies. Due to the challenges associated with sorting mixed waste, directly processing waste streams offers a practical approach. Polyethylene terephthalate (PET) and high-density polyethylene (HDPE) are common consumer plastics, but they are difficult to recycle together due to immiscibility and degradation. In mixed waste, recycled HDPE (r-HDPE) often contaminates the recycled PET (r-PET) stream. Additive manufacturing (AM) offers a promising solution to upcycle these mixed polymers into functional products with minimal waste. This study investigates the processing and characterization of r-PET/r-HDPE blends for AM, focusing on the role of compatibilizers in enhancing their properties. Blends were melt-compounded using a twin-screw extruder to improve dispersion, followed by direct pellet-based 3D printing. A compatibilizer (0–7 php) was incorporated to improve miscibility. Rheological testing showed that the 5 php compatibilizer optimized viscosity and elasticity, ensuring smoother extrusion. Thermal analysis revealed a 30 °C increase in crystallization temperature and a shift in decomposition temperature from 370 °C to 400 °C, indicating improved thermal stability. Mechanical testing showed a tensile strength of 35 MPa and 17% elongation at break at optimal loading. Scanning electron microscopy (SEM) confirmed reduced phase separation and improved morphology. This work demonstrates that properly compatibilized r-PET/r-HDPE blends enable sustainable 3D printing without requiring polymer separation. The results highlight a viable path for the conversion of plastic waste into high-value, customizable components, contributing to landfill reduction and advancing circular economy practices in polymer manufacturing.

## 1. Introduction

Despite sustainability regulations and environmental concerns, the usage rate of plastic for packaging industries and consumer products is steadily increasing. In Canada, over 3 million tonnes of plastic waste are generated, of which 9% is recycled and the rest ends up in landfills, posing a significant threat to the environment, including oceans, lakes, and rivers [1]. More importantly, it is predicted that the amount of plastic waste in the ocean will exceed the mass of the global stock of fish by 2050 [2]. Over the past 70 years, the global production of conventional plastics (excluding fiber-reinforced composites) has expanded dramatically, reaching 359 million metric tons (Mt) in 2018, driven by their versatile applications and cost-effectiveness [3]. Despite their widespread use, plastic recycling remains limited, with 79% accumulating in landfills or leaking into the environment, including oceans [1,4,5,6]. Plastic packaging constitutes almost half of all global plastic waste, including polyethylene terephthalate (PET), high-density polyethylene (HDPE), and polypropylene (PP) [7,8]. For instance, in 2015, 141 Mt of plastic packaging waste was generated globally, with recycling rates as low as 14%. Improper disposal has severe consequences, particularly for ocean ecosystems, where approximately 4.8 to 12.7 Mt of plastics enter annually from coastal regions [9,10]. The degradation of larger plastics into microplastics further exacerbates the problem, posing risks to marine life and humans through bioaccumulation in food chains.

To change this trend, there is an urgent need to develop sustainable manufacturing techniques and strategies to tackle the above-mentioned issue. One of the main obstacles to recycling plastic is the sorting process. Waste streams often contain different types of plastics—like yogurt tubes with PP bodies and HDPE caps, or water bottles with PET bodies and HDPE or LDPE lids—which account for around 10% of total plastic waste [11]. As a result, the production of recycled polymers is often not cost-competitive with that of virgin products. One potential solution is to prepare polymer blends from the stream of waste and reuse the waste mix as new feedstock, thereby lowering costs and reducing the need for sorting [12,13,14,15]. In addition, through polymer blending, there is an opportunity to tailor the mechanical and rheological properties of the blend based on the final application.

Among the plastics that are utilized in the packaging industry, PET and HDPE are used extensively for the manufacturing of water bottles and bottled beverages [16,17]. Instead of separating HDPE caps from PET bodies, they can be blended with each other through the melt-extrusion process; however, these polymers are immiscible, leading to poor interfacial adhesion, which can negatively affect the Physico-mechanical properties. The compatibility of these mixed polymer systems can be improved through various approaches, including reactive and non-reactive methods [18,19,20,21]. Typically, graft or block copolymers are used to facilitate this compatibility [20,21,22,23]. These copolymers can interact physically or chemically with the blend’s components, thereby lowering interfacial tension and enhancing phase dispersion and adhesion through interpenetration and entanglement at the polymer interfaces [18,23,24,25]. In the case of reactive compatibilization, copolymers are formed in situ during the melt-blending process by using polymers that contain functional groups—such as carboxyl, anhydride, or epoxy—which can react with the other polymer components.

The utilization of waste plastics for material extrusion-based AM, including fused filament fabrication (FFF), has been explored in the literature. Zander et al. [26] investigated the feasibility of using recycled polypropylene (rPP) blends for FFF applications. Their study focused on rPP blended with recycled polyethylene terephthalate (rPET) and recycled polystyrene (rPS) with and without compatibilizers. The authors reported that rPET exhibited the highest tensile strength among the tested materials, reaching 35.1 MPa. The study also highlighted improvements in elongation at break for the blended materials compared to neat rPET and rPS, making them more suitable for AM applications. Specifically, the elongation at break for printed rPET was 60%, whereas rPP exhibited 14%. The rPP/rPET 50-50 blend without a compatibilizer had an elongation at break of 47%, while the SEBS-compatibilized blend reached 23.1%, and the SEBS-MA blend reached 24.2%. In contrast, rPS exhibited brittle behavior with an elongation at break of less than 1%. In another study, Rocha et al. [27] examined novel ABS-based binary and ternary polymer blends for material extrusion 3D printing. Their research focused on blending ABS with SEBS and ultrahigh-molecular-weight polyethylene (UHMWPE) to enhance mechanical performance. The results showed that blends with 20% and 50% SEBS experienced reduced ultimate tensile strength (UTS) but exhibited increased elongation at break. Specifically, the 50:50 ABS–SEBS blend demonstrated an elongation at break of 47.6%, compared to 8.6% for pure ABS. Additionally, blends incorporating UHMWPE and SEBS showed improved surface finish, making them potential candidates for FFF applications.

In addition to compatibilized polymer blends, several recent studies have investigated the reinforcement of recycled thermoplastics with fibers to improve mechanical performance in additive manufacturing and injection molding applications. These approaches highlight the growing interest in upcycling waste materials into functional composites suitable for 3D printing and other fabrication methods [28,29,30].

Vaucher et al. [28] explored the effect of high-density polyethylene (HDPE) contamination on the microstructure and mechanical properties of 3D-printed recycled PET. Their study demonstrated that up to 5 wt% HDPE could be incorporated into recycled PET without significant detriment to print quality. However, higher HDPE content resulted in phase separation and reduced mechanical performance. In addition, they did not use a compatibilizer for this blend, which deteriorated the properties. In another study, Gonzalez et al. [29] investigated multi-material distributed recycling via material extrusion using a blend of PET and HDPE. Instead of utilizing melt compounding before printing, the authors manually mixed shredded PET and HDPE flakes and directly fed them into a pellet-based 3D printer. This method resulted in heterogeneous mixing, leading to variations in material properties. The authors successfully printed a functional chair with a reduction in production costs of up to 88%.

Our study tackles the challenges associated with processing r-PET/r-HDPE blends derived from mixed waste streams by using suitable compatibilizers. We fully characterized and analyzed the physico-mechanical properties of these polymer blends to better understand how the incorporation of appropriate additives can play a critical role in advancing practical plastic recycling applications. Unlike the previous studies that primarily relied on the manual mixture of polymers before direct 3D printing—leading to heterogeneous phase dispersion and poor properties—this study employs melt compounding via a twin-screw extruder to ensure uniform dispersion of the recycled polymers and compatibilizers. The composition of recycled bottles consists primarily of PET for the body and HDPE for the caps, with an approximate weight ratio of 90% PET to 10% HDPE. This ratio is selected based on common bottle manufacturing practices, where the cap typically constitutes around 8–10% of the total bottle weight. By optimizing compatibilizer concentrations (0, 2, 5, and 7 php), this study systematically investigates its effect on the interfacial adhesion, mechanical integrity, and printability of recycled PET/HDPE. The results demonstrate that a 5 php compatibilizer loading offers the best balance between mechanical properties and processing stability. The use of pelletized composite feedstock rather than manually mixing separate polymer flakes improves the homogeneity of the final printed structure, addressing the printability limitations observed in prior research. To comprehensively evaluate the developed materials, a rheological analysis was performed to assess viscosity and flow behavior, a thermal analysis was conducted to examine crystallization behavior and thermal stability, tensile tests were carried out to determine mechanical performance, and scanning electron microscopy (SEM) was used to analyze the morphology and phase dispersion of the polymer blends.

By refining material formulations and processing techniques, this work advances the sustainable application of recycled PET-HDPE blends in additive manufacturing, ensuring superior phase compatibility and mechanical performance compared to existing approaches.

## 2. Materials and Methods

### 2.1. Materials

Recycled PET flakes (r-PET), sourced from post-industrial water bottle waste, were purchased from Post Plastics Inc Canada (flake size: 3 mm) [31]. The recycled HDPE pellets were purchased from Oceanworks, a company in the US (pellet size: 2 mm) [32]. In addition, the blended pellets’ size was approximately 3 mm. Due to ongoing patenting, the exact chemical composition and structure of the compatibilizer used in this study remain confidential.

### 2.2. Polymer Blending with Twin-Screw Extruder

Prior to mixing, all r-PET flakes, r-HDPE, and compatibilizer pellets were dried in an oven at 70 °C for 12 h. All sample pellets were manually mixed for 10 min before being fed into the twin-screw extruder hopper. For melt blending, a Leistritz ZSE18HP-400 co-rotating twin-screw extruder was used, with heating zones set between 190 and 260 °C and a screw speed of 400 rpm. After blending and mixing in the extruder’s barrels, the molten polymer blend was passed through a cold-water bath and drawn out through a pelletizer. The solidified polymers were then chopped into small pellets. Before printing, the prepared pellets were re-dried in the oven at 70 °C for another 12 h to remove any remaining moisture.

### 2.3. Pellet 3D Printing

**Figure 1:**
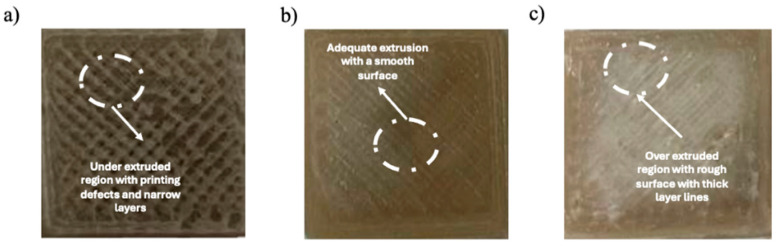
3D-printed test samples for flow rate troubleshooting (**a**) under extrusion, (**b**) proper extrusion, and (**c**) over extrusion.

To streamline the additive manufacturing (AM) process for the blended polymer, the conventional step of producing filament using a single-screw extruder and subjecting it to an additional heating cycle for FFF printing was bypassed. This reduced thermal degradation, enhanced process efficiency, and eliminated the variability associated with filament extrusion. Instead, a customized direct pellet 3D printer was employed, allowing direct printing from pellet feedstock, thereby simplifying material handling and optimizing print quality (Figure 1).

### 2.4. Printer Configuration

A V4 pellet extruder (Mahor Company, New York, NY, USA) was mounted on an aluminum gantry, serving as the primary extrusion system. The extruder was integrated with modified Marlin firmware, which was adjusted to accommodate the pellet-based extrusion mechanism. The firmware modification was necessary because standard configurations are typically optimized for filament-based fused filament fabrication (FFF) rather than pellet extrusion.

**Figure 2:**
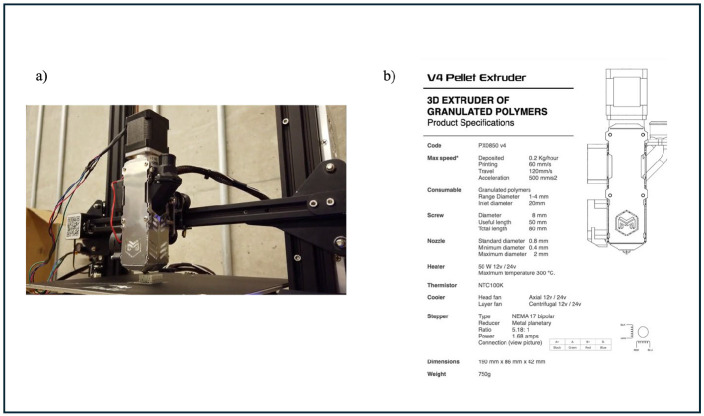
(**a**) Customized pellet 3D printer; (**b**) pellet extruder specification.

The V4 extruder operates using a single-screw mechanism with one heating zone, as illustrated in Figure 2. The screw-driven system ensures that polymer pellets are gradually melted, homogenized, and extruded through the nozzle under controlled temperature and pressure conditions. However, due to the inherent differences between pellet extrusion and filament-based systems, optimizing flow rate and material deposition posed a significant challenge.

To calibrate material flow and ensure consistent deposition, the slicing software Slic3r (1.3.0) was used. However, as the firmware was originally designed for filament extrusion, the extrusion multiplier required fine-tuning. A range of extrusion multipliers (4 to 5) was tested. The key observations were as follows:

Multiplier = 5 → Resulted in over-extrusion, causing excess material deposition and poor dimensional accuracy.

Multiplier = 4 → Led to under-extrusion, reducing interlayer adhesion and weakening mechanical properties.

Multiplier = 4.5 → Provided an optimal balance, ensuring uniform flow, proper layer bonding, and structural integrity of printed samples.

Temperature regulation was a critical factor in achieving stable material extrusion, as the single heating zone in the V4 extruder was solely responsible for melting the polymer. The barrel was divided into the following functional zones:

Feeding Zone → The temperature must be high enough to soften the polymer pellets and allow the screw to rotate efficiently.

Melting and Mixing Zone → The middle section of the barrel ensures the homogeneous blending of r-PET, r-HDPE, and the compatibilizer.

**Figure 3:**
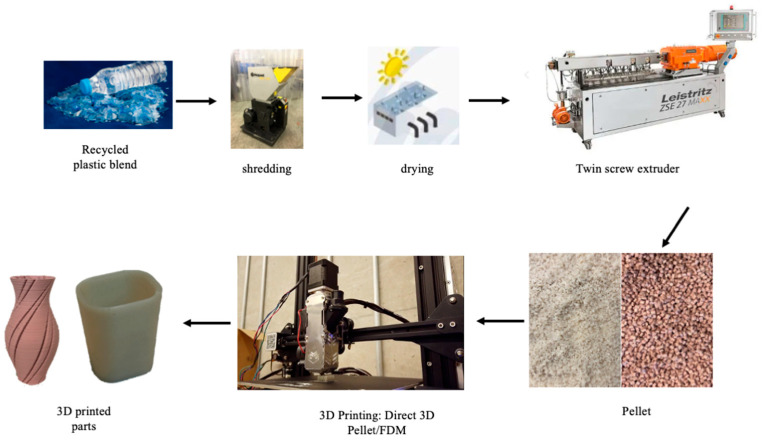
Process schematic.

During testing, it was observed that nozzle temperatures between 260 °C and 295 °C frequently caused screw clogging, disrupting the printing process. This occurred due to the incomplete melting of the pellets, leading to inconsistent flow and extrusion failure. However, setting the nozzle temperature to 300 °C provided optimal performance, preventing clogging at the feeding zone while maintaining a stable melt viscosity. The process schematic is illustrated in Figure 3.

## 3. Characterization

### 3.1. Rheology Test

To examine the material’s flow properties, a thorough rheological study was carried out at a temperature of 260 °C using a parallel plate rheometer (DHR, TA Instruments, USA, New Castle, DE, USA). The rheometer setup featured 25 mm diameter parallel plates with a controlled gap of 1500 µm, ensuring precise measurements and uniform stress distribution during testing. The material samples were prepared as disks, each 25 mm in diameter and 2 mm thick. These dimensions were chosen to match the parallel plate geometry and promote even stress application across the sample. Careful preparation of the samples was key to obtaining reliable and consistent rheological data. A frequency sweep was performed, with shear frequencies varying from 1 to 100 rad/s, to capture the material’s behavior across a wide range of shear rates. The experiment was conducted at a constant strain rate of 5%, ensuring that the test remained within the linear viscoelastic region of the material. This approach guaranteed that the material’s response to applied stress was linear, providing reliable data for its rheological characterization.

### 3.2. Thermal Characterization

To analyze the thermal properties of r-PET/r-HDPE blend, differential scanning calorimetry (DSC) measurements were carried out using a Q20 instrument from TA Instruments (New Castle, DE, USA). Testing was performed in a nitrogen environment with a flow rate of 50 mL/min, and samples weighing approximately 6 mg were used. To remove any prior thermal history, each sample underwent three cycles—heating, cooling, and reheating. The heating process reached up to 300 °C, followed by a 3 min isothermal hold. Cooling was then applied at a rate of 10 °C/min down to 30 °C, followed by a second heating stage at 10 °C/min, again reaching 300 °C. From the DSC data, the melting temperature ($T_{m}$), cold crystallization temperature ($T_{c}$), glass transition temperature ($T_{g}$) were extracted. Thermogravimetric analysis (TGA) was performed using a Q50 analyzer from TA Instruments (USA) to determine the decomposition temperature and quantify the residue content in the r-PET blend samples. Samples, with an average weight of 5 mg, were analyzed in a nitrogen-controlled atmosphere. The temperature ranged from 30 °C to 600 °C, increasing at a rate of 10 °C/min.

### 3.3. Mechanical Test

**Figure 4:**
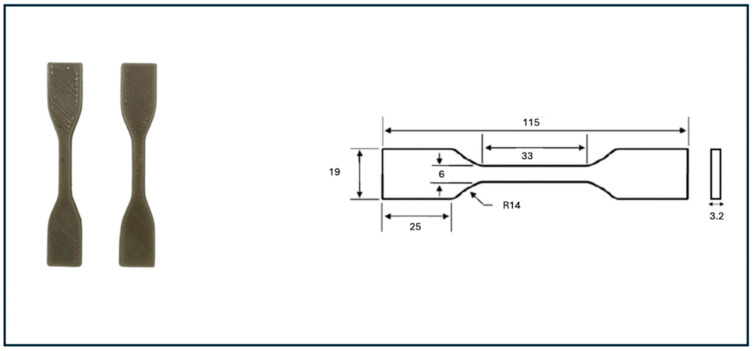
Tensile test samples printed based on ASTM D638.

Tensile testing of Type IV dog-bone specimens (with raster angle of −45/+45)—3D printed to meet ASTM D638 standards [33]—was conducted using a high-precision Universal Testing Machine (LS100 Plus, Lloyd, UK) with a 100 kN load capacity [33]. Specimens (Figure 4) were clamped securely on flat supports and aligned precisely along the machine’s central axis to ensure accurate measurement. Testing was performed at a constant crosshead speed of 5 mm/min, allowing for the reliable evaluation of mechanical properties. To capture accurate strain data, an extensometer was applied to each sample during testing. Three specimens were printed and tested for each formulation. The images and dimensions of the printed samples are provided in the results section.

### 3.4. SEM Analysis

To analyze the fracture surface of the blended pellets, SEM (Flex SEM 1000, Hitachi, Japan) analysis was carried out. To obtain a conductive surface, all samples were coated with a thin layer of gold. SEM analysis was crucial for evaluating the blending process and the effect of additives on the morphology of the polymer.

## 4. Results and Discussion

### 4.1. Rheology Test

**Figure 5:**
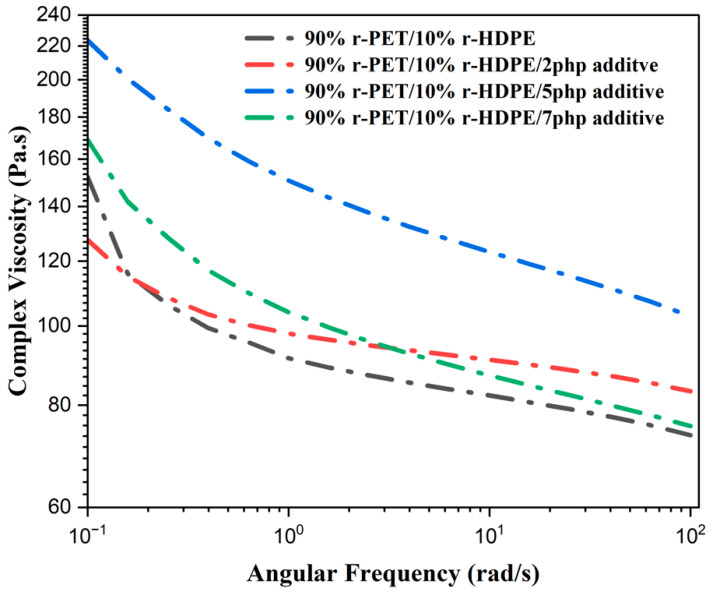
Complex viscosity of r-PET/r-HDPE samples modified with compatibilizer.

**Figure 6:**
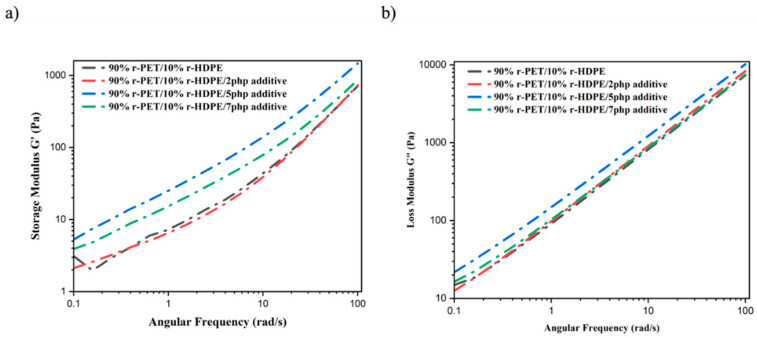
r-PET/r-HDPE samples modified with compatibilizer: (**a**) storage modulus; (**b**) loss modulus.

The complex viscosity, storage modulus (G′), and loss modulus (G″) of all r-PET/r-HDPE blend samples, both modified and unmodified, are presented in Figure 5 and Figure 6. As seen in the graphs, all samples exhibit shear-thinning behavior, where viscosity decreases with increasing shear rate. This property is favorable for extrusion-based 3D printing, as it facilitates smooth material flow through the nozzle while ensuring sufficient structural integrity upon deposition. By adding 2 and 5 php compatibilizer, a significant increase in complex viscosity was observed. This change improved clarity and accuracy in describing the experimental formulation, compared to the pure r-PET/r-HDPE blend. The primary reason for this viscosity enhancement is attributed to stronger intermolecular interactions between r-HDPE, r-PET, and the functional groups of the additive, which facilitate improved interfacial adhesion and dispersion [30]. The presence of a compatibilizer reduces coalescence suppression and interfacial tension, allowing the HDPE phase to disperse more evenly within the PET matrix. This leads to a morphological transition from a heterogeneous blend with discrete HDPE domains to a smoother and more continuous phase, thereby improving viscosity and interfacial bonding [30]. This viscosity enhancement is beneficial for extrusion-based 3D printing, as it promotes better shape retention, improves interlayer bonding, and reduces the risk of flow instability during deposition.

The storage modulus (G′) and loss modulus (G″) (Figure 6a,b) follow a similar trend, showing increased values with the addition of 2 php and 5 php compatibilizer. The rise in G′ indicates enhanced elasticity and stiffness due to better load transfer across the phases, while the increase in G″ reflects improved energy dissipation and viscoelastic balance. These results suggest that the addition of a compatibilizer results in a more structured polymer network, enhancing both elastic and viscous properties, which is beneficial for mechanical integrity in 3D-printed components.

However, when the compatibilizer content exceeded 5 php, the complex viscosity, storage modulus, and loss modulus decreased. This can be attributed to phase plasticization, molecular entanglement reduction, and possible phase separation. Beyond a critical compatibilizer concentration, excess molecules may accumulate within the continuous phase, leading to localized softening. This, in turn, disrupts the polymer entanglement network and effectively reduces viscosity [11,31,34]. Additionally, surplus compatibilizer may cause self-association rather than interfacial interaction, leading to aggregation and phase separation, which prevents uniform stress transfer across the material and weakens the structural integrity [30]. This justifies why adding more than 5 php compatibilizer does not provide further improvement and, in some cases, results in weakened interfacial adhesion and rheological performance. These findings confirm that 5 php compatibilizer is the optimal concentration for achieving enhanced viscosity, elasticity, and phase interaction in 90%r-PET/10%r-HDPE blends for 3D printing applications. Beyond this threshold, the adverse effects of phase separation, interfacial saturation, and molecular instability become dominant, limiting further performance enhancements.

### 4.2. Differential Scanning Calorimetry

**Figure 7:**
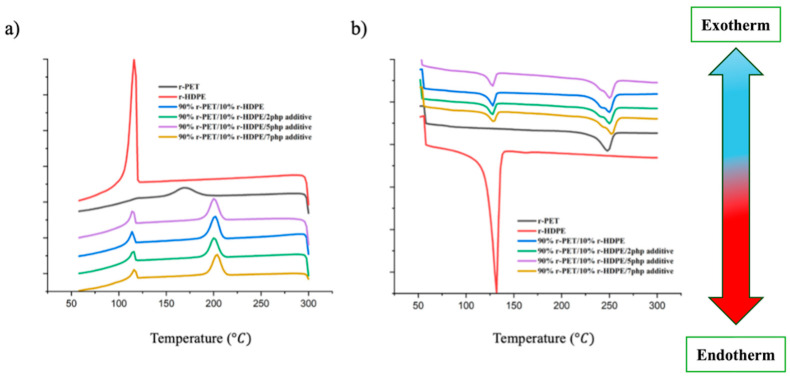
(**a**) Cooling and (**b**) heating curves of modified samples with compatibilizer.

The differential scanning calorimetry (DSC) analysis was conducted to evaluate the thermal transitions of the r-PET/r-HDPE blends modified with a compatibilizer during both heating and cooling cycles. This analysis provides insights into the melting behavior, crystallization kinetics, and phase interactions within the blend. The key thermal parameters assessed include the melting temperature ($T_{m}$) and crystallization temperature ($T_{c}$), which are essential for understanding the compatibility and structural evolution of the polymer blend (Table 1) (Figure 7).

During the heating cycle (Figure 7b), the melting temperature ($T_{m}$) of r-PET increased by 3 °C upon the addition of the compatibilizer (from 247 to 250). This increase in $T_{m}$ suggests that the compatibilizer improves the crystalline order of r-PET, likely by enhancing phase miscibility and reducing chain mobility in the amorphous regions. The presence of a compatibilizer may act as an interfacial agent, restricting r-HDPE molecular motion and promoting more ordered crystalline structures within the r-PET phase. This behavior is consistent with previous studies on compatibilized PET/polyolefin blends, where compatibilizers facilitated interfacial interactions, leading to an increase in $T_{m}$ due to the formation of stronger crystalline regions [35,36]. In contrast, the melting temperature of r-HDPE remained relatively unchanged, indicating that the compatibilizer predominantly affects the r-PET phase rather than the r-HDPE phase. The small exothermic peak near 120 °C observed in the cooling curve of r-PET is attributed to the recrystallization of cold segments or low-molecular-weight oligomers in the recycled PET matrix. This phenomenon is often observed in recycled polymers with heterogeneous chain lengths or partial degradation [37].

The cooling cycle (Figure 7a) reveals significant changes in the crystallization temperature ($T_{c}$) of r-PET in compatibilized blends. The addition of compatibilizer caused an increase in $T_{c}$, from 175 to around 196 °C, suggesting enhanced nucleation efficiency due to the presence of interfacial interactions and phase refinement. The increase in $T_{c}$ is advantageous in processing, as it leads to a shorter solidification time, which can improve dimensional stability and reduce processing cycles in polymer manufacturing [35,38].

**Figure 8:**
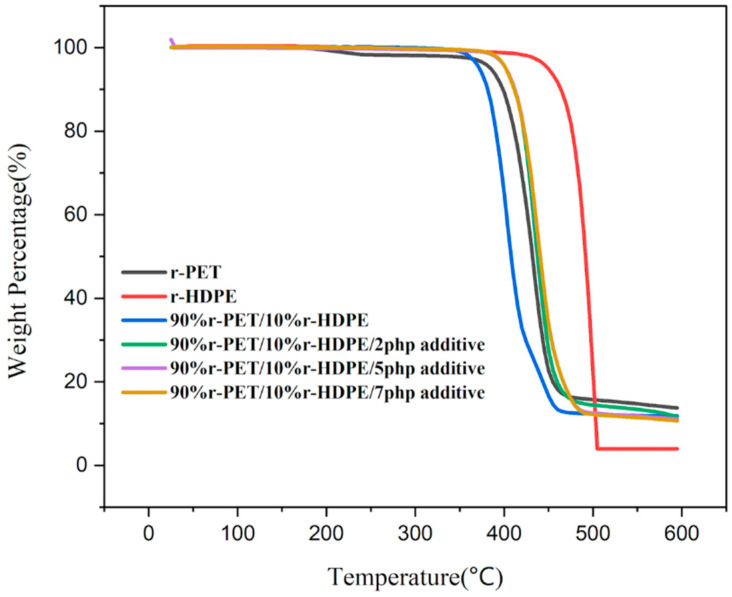
TGA test results.

From the TGA analysis (Figure 8), the degradation onset temperature for r-PET is observed at approximately 390 °C, while r-HDPE decomposes at a higher temperature of around 450 °C. The higher thermal stability of r-HDPE can be attributed to its molecular structure, consisting of long-chain saturated hydrocarbons, which require higher thermal energy to break down [18,19,20,32]. The char residue at the end of the TGA test is notably higher for r-PET compared (around 13%) to r-HDPE. This increase in residual mass can be linked to additives and contaminants introduced during the previous manufacturing processes, which can include flame retardants, stabilizers, or fillers commonly found in PET-based materials.

After blending 10% r-HDPE with r-PET, the decomposition temperature decreases to around 370 °C. This reduction is likely due to phase incompatibility between r-PET, a polar polyester, and HDPE, a nonpolar polyolefin, leading to interfacial defects that facilitate thermal degradation at lower temperatures. The incorporation of 2–7 php of a compatibilizer effectively shifts the decomposition temperature back to approximately 400 °C. This increase in thermal stability suggests that the compatibilizer enhanced interfacial adhesion, reducing phase separation, and improving the blend’s thermal resistance [20,21]. The improved dispersion of r-HDPE within the r-PET matrix likely delays degradation by restricting polymer chain mobility and stabilizing the material at elevated temperatures.

### 4.3. Mechanical Properties

**Figure 9:**
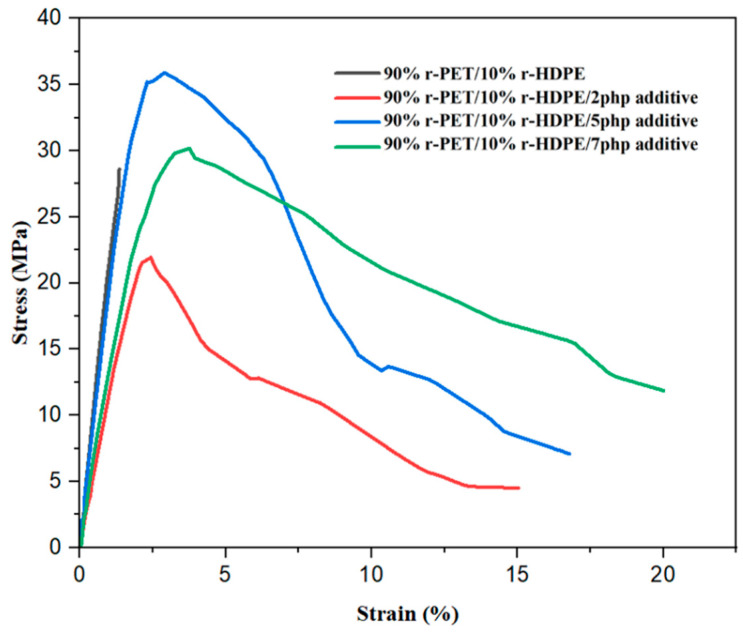
Mechanical properties of compatibilized blends.

The stress–strain curves of the r-PET/r-HDPE blends modified with different levels of compatibilizer provide insights into the effects of these additives on mechanical performance. The compatibilizer plays a crucial role in improving interfacial adhesion (Figure 9).

Although the unmodified blend showed the lowest elongation at break (~1.1%), it did not exhibit the lowest tensile strength. In fact, the blend with 2 php compatibilizer had a slightly lower UTS (~22 MPa), likely due to partial compatibilization without sufficient interfacial strength. At 5 php compatibilizer, a significant increase in tensile strength and strain at break was observed, indicating improved stress transfer and enhanced interfacial bonding, with around 35 MPa UTS and 17% elongation at break. The sample with 7 php compatibilizer, however, exhibited a reduction in UTS (30 MPa) and 22.5% elongation at break, suggesting that excessive compatibilizer may lead to phase plasticization, reducing the stiffness of the blend [23,39,40,41]. This behavior aligns with previous studies on compatibilized PET/polyolefin blends, where excess compatibilizer resulted in reduced mechanical performance due to phase saturation [33].

### 4.4. Scanning Electron Microscopy Analysis

**Figure 10:**
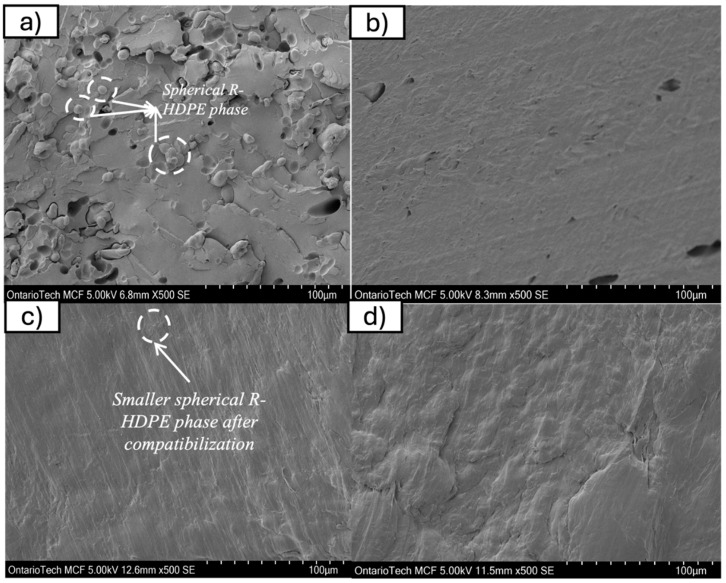
(**a**) Raw PET-HDPE, (**b**) PET-HDPE with 2 php compatibilizer, (**c**) PET-HDPE with 5 php compatibilizer, (**d**) PET-HDPE with 7 php compatibilizer.

To investigate the effect of compatibilizer on the morphology of the blend, SEM analysis was conducted on samples at different magnifications to observe the r-HDPE and r-PET phases. In unmodified samples, the r-HDPE phase was revealed in a spherical form (Figure 10a)—a typical morphology in immiscible polymer blends due to thermodynamic incompatibility, where one polymer forms a dispersed phase within the continuous phase of the other. The spherical shape is primarily a result of interfacial tension minimization, as spheres have the lowest surface area-to-volume ratio, reducing the interfacial free energy between the two polymers. Additionally, phase separation dynamics during melt processing led to the breakup and coalescence of the dispersed phase, where shear forces shape the minor phase into droplets before the morphology is “frozen” in place [22,42,43].

Moreover, viscosity and elasticity effects play a crucial role, as differences in the viscosity of the two phases influence morphology; a lower-viscosity dispersed phase promotes spherical droplet formation under shear flow. However, after the addition of the compatibilizer, very little r-HDPE phase was observed in the SEM images, suggesting that the compatibilizer effectively improved interfacial adhesion, leading to finer dispersion and enhanced integration of r-HDPE within the r-PET matrix. This reduction in distinct r-HDPE domains can be attributed to enhanced interfacial interactions, which lower the interfacial tension and prevent the coalescence of r-HDPE droplets, thereby creating a more homogeneous blend [22,42,43,44,45].

## 5. Conclusions

This study presents a systematic approach to enhancing the compatibility and mechanical performance of r-PET/r-HDPE blends for additive manufacturing applications. By employing melt compounding via twin-screw extrusion and incorporating the appropriate compatibilizer at varying concentrations, we addressed key challenges in immiscible polymer blending. The findings of this research are summarized as follows:

### 5.1. Rheological Analysis

- The addition of compatibilizer up to 5 php significantly increased complex viscosity, storage modulus (G′), and loss modulus (G″), enhancing elasticity and interfacial adhesion.
- Excess compatibilizer (>5 php) led to a decrease in viscosity and modulus due to phase plasticization and interfacial saturation.

### 5.2. Thermal Characterization

- The results of the differential scanning calorimetry (DSC) analysis showed that the compatibilizer increased the melting temperature (Tm) of r-PET, improving crystallinity and phase miscibility.
- The crystallization temperature (Tc) of r-PET increased with compatibilizer addition, leading to improved nucleation efficiency and faster solidification.
- Thermogravimetric analysis (TGA) demonstrated enhanced thermal stability with compatibilizer addition, shifting the decomposition temperature of the blend from 370 °C to approximately 400 °C.

### 5.3. Mechanical Properties

- The unmodified r-PET/r-HDPE blend exhibited poor mechanical properties due to weak interfacial adhesion (28 MPa UTS and 3% elongation at break).
- The addition of 2 php compatibilizer increased elongation at break to 15% but reduced tensile strength, suggesting limited compatibility improvements.
- At 5 php compatibilizer, the blend exhibited the best mechanical performance, with 35 MPa UTS and 17% elongation at break, demonstrating improved stress transfer and interfacial bonding.
- The 7 php compatibilizer blend exhibited phase plasticization effects, resulting in a UTS reduction to 30 MPa but increased elongation at break to 22.5%, suggesting a shift in balance between stiffness and ductility.

### 5.4. Morphological Analysis (SEM)

- Unmodified r-PET/r-HDPE blends exhibited phase separation with distinct HDPE domains, indicating poor compatibility.
- Compatibilizer addition resulted in finer dispersion of HDPE within the PET matrix, reducing interfacial defects and improving blend homogeneity.
- At 5 php, the morphology transitioned to a more uniform structure with enhanced adhesion, while 7 php led to self-aggregation effects.

### 5.5. 3D Printability & Process Optimization

- Direct pellet extrusion printing was optimized to improve flow consistency and eliminate nozzle clogging.
- A calibrated extrusion multiplier (4.5) was found to provide the best balance between flow rate and layer adhesion.
- Optimized nozzle temperature (300 °C) ensured stable extrusion, preventing incomplete melting and phase separation.

## Figures and Tables

**Table 1:** Data extracted from DSC test.

Sample	Tm (°C)	Tc (°C)
r-PET	247.0	175
r-PET/r-HDPE (no compatibilizer)	248.0	203
r-PET/r-HDPE + 2 php	248.5	201
r-PET/r-HDPE + 5 php	250.0	204
r-PET/r-HDPE + 7 php	252	206

## Data Availability

The original contributions presented in this study are included in the article. Further inquiries can be directed to the corresponding author.
